# The Transplantation of Pancreatic Islets to Portal Vein: The Influence on Liver Tissue

**DOI:** 10.3390/ijms27031419

**Published:** 2026-01-30

**Authors:** Alžběta Vojtíšková, Eva Fábryová, Zuzana Berková, Tomas Koblas, Peter Girman, Jan Kříž

**Affiliations:** 1 Center of Experimental Medicine, Institute for Clinical and Experimental Medicine, 140 21 Prague, Czech Republic; alvj@ikem.cz (A.V.); vode@ikem.cz (E.F.); zube@ikem.cz (Z.B.); tokb@ikem.cz (T.K.); 2 1st Faculty of Medicine, Charles University, 128 53 Prague, Czech Republic; 3 Diabetes Center, Institute for Clinical and Experimental Medicine, 140 21 Prague, Czech Republic; pegi@ikem.cz

**Keywords:** pancreatic islet, liver, side effect, insulin overstimulation, cystic lesions, thrombosis, portal vein, perfusion impairment

## Abstract

Pancreatic islet (PI) transplantation (Tx) to the portal vein is an established therapeutic modality for selected type 1 diabetic patients. However, a comprehensive review considering the effects of PIs on surrounding liver tissue is lacking. Typical interactions can be detected in the early and delayed phases. This review summarizes known side effects of PI transplantation. In early phase the interaction occurs immediately upon contact of the PI into portal vein blood. Mechanical obstruction, exacerbated by thrombosis as part of the instant blood-mediated inflammatory reaction (IBMIR), leads to ischemic injury to adjacent liver tissue. Delayed changes, such as focal steatosis and glycogen accumulation appear days to weeks after Tx and are caused by local overstimulation of hepatocytes by insulin in supraphysiological concentrations. In animal models these lesions could progress over months to cystic cholangiomas or hepatocellular carcinomas. Such neoplastic changes have been observed in experimental animals; they have not been reported in human patients. In conclusion, while PITx into the liver is not an optimal procedure, it currently represents the site offering the best functional integration of the graft. The adverse effects discussed are pronounced but generally not severe, nor do they appear to compromise the overall health status of the recipients.

## 1. Introduction

Insulin injections are essential for diabetic patients who do not produce this hormone naturally. However, they rarely enable patients to achieve long-term metabolic normalization. The transplantation of insulin-producing pancreatic islets is a promising therapeutic alternative that involves the isolation of islets from donor pancreas and subsequent infusion into the liver via the portal vein. While this technique is well-founded and has the potential to restore adequate insulin production, careful attention to dosing and placement of the graft can minimize the risk of temporarily elevated local insulin levels within tissue surrounding the grafts. In this review, we summarize the key pathophysiological consequences of pancreatic islet transplantation, focusing particularly on the potential side effects arising from the direct interactions between transplanted islets and liver tissue and the overall safety of the procedure.

### 1.1. Insulin-Dependent Diabetes Mellitus and Therapeutic Context

In the case of insulin-dependent diabetes, the hormone can be replaced by subcutaneous injections that in principle cannot be absolutely precise. The only active insulin is that which is already present in blood. There is naturally a minimal delay between the secretion of insulin from the ß-cell and its entering the capillary blood, but when administered subcutaneously, the maximum insulin concentration in the blood is not reached before one hour [1]. To address this shortcoming, many insulin modifications have been developed to allow both a very smooth and gradual release from the subcutaneous deposit to cover basal needs and rapid, short-term release to cover prandial needs. The most modern therapeutic approach is the continuous administration of a fast-acting insulin analog via a pump controlled by an algorithm based on continuous monitoring of glucose concentration in the interstitial subcutaneous fluid [2,3].

The most cases of insulin-dependent diabetes mellitus (IDDM) are attributable to two primary pathogenic mechanisms. **Type 1** diabetes is characterized by the autoimmune destruction of pancreatic β-cells, leading to a total deficiency in insulin production that manifests over several months or years. **Type 3c** diabetes mellitus is of iatrogenic origin, arising as a consequence of total pancreatectomy. The condition is characterized by the absence of digestive enzymes and the prompt cessation of endogenous insulin secretion, resulting in the inability to regulate blood glucose levels adequately. This also determines the primary indications for transplant treatment. Pancreas organ transplantation has become a very successful and safe treatment technique. The main limitation is the shortage of suitable organs. However, thanks to transplant teams’ extensive experience, more than 90% of patients achieve independence from exogenous insulin treatment. Although all islets are transferred and the graft’s vascular system is immediately connected, complications can arise from the exocrine tissue.

For allogeneic islets, the indication is the same, namely type 1 diabetes combined with end-stage diabetic kidney disease and cardiovascular limitations that prevent pancreas organ transplantation. The islets are isolated from pancreas of non-diabetic cadaveric donors for this purpose. For autologous islets, the indication is total pancreatectomy due to non-malignant causes. The surgical intervention can be enhanced by the islet isolation from their own excised pancreas and subsequent transplantation to the portal vein. The optimal outcome can be considered prevention of diabetes development.

### 1.2. Pancreatic Islets—Special Endocrine Micro-Organs

**Figure 1:**
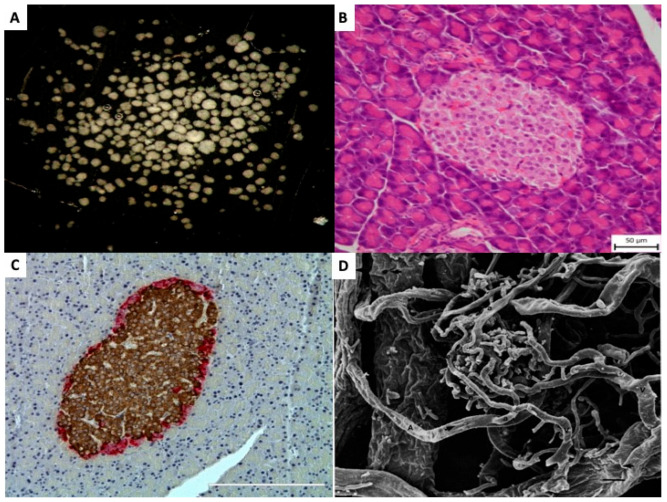
**Pancreatic islets** (**A**) Isolated pancreatic islets in black dish, ready for handpicking (×20). (**B**) Light microscopic (Hematoxillin and Eosin) picture of pancreatic islet within tissue of exocrine pancreas (×200). (**C**) Light microscopic (Immunohistochemistry) picture of pancreatic islet within pancreas. Brown color indicates insulin and red color indicates glucagon presence in the islet cells (×200). (**D**) Scanning electron micrography of a corrosion cast of pancreatic vessels. Arterioles (A) and venules (V) can be identified and distinguished by the different imprints. Numerous efferent capillaries (a) arise from this glomerulus, pass around the islet, and coalesce into collecting venules. (×490). Published by John Wiley & Sons Ltd. [9].

Islet transplantation is unique among other types of transplantation. It is more complex than the transplantation of individual cells, such as in bone marrow transplants, and the graft is not directly reconnected to the host’s bloodstream by the surgeon, as in whole organ transplants. Pancreatic islets (=Islets of Langerhans) are small, dispersed, micro-organs consisting (Figure 1A) of up to several thousand cells, Figure 1B. Morphologically they consist of at least five distinct endocrine cell types, as well as blood, vascular, stromal, immune, and neural cell populations [4,5,6], Table 1. Although the precise cellular composition varies depending on islet size, anatomical location, and species, insulin-producing β-cells represent the most abundant endocrine cell type [7], Figure 1C [8].

Each endocrine cell is in direct contact with a capillary wall, and the density of the capillary network within the islets is 5- to 10-fold greater compared to surrounding exocrine tissue. Its specific structure varies depending on islet size [9]. In small islets (diameter < 160 µm), the capillaries are integrated with the exocrine capillary network, forming a glomerulus-like insulo-acinar system. In contrast, large islets (diameter > 250 µm) are typically supplied by 1 to 5 arterioles originating from intralobular arteries. These arterioles branch into dense fenestrated capillaries—comprising approximately 7–8% of the islet volume—which drain directly into venules, forming an insulo-venous system [7,9,10]. A representative example of large islet microvasculature is illustrated in Figure 1D, using scanning electron microscopy of a corrosion cast [9].

Although pancreatic islets account for only 1–2% of the total pancreatic volume, they receive nearly 20% of the whole organ blood supply [9,11,12]. Perfusion studies have identified three distinct microcirculatory flow patterns within islets in mice, which allows for the paracrine interaction among cells [13].

Overall, islet blood flow is approximately 5–6 mL/min/g of pancreas, which contrasts sharply with the lower perfusion rate of exocrine tissue (0.4–1 mL/min/g). This high perfusion rate enables the immediate dilution and efficient transport of insulin released from β-cells towards the portal vein [7,14]. Interestingly, the acinar cells located adjacent to islets exhibit notable morphological differences compared to those located farther away. They tend to be larger and often polynuclear, as well as containing more secretory granules [15,16,17]. This exceptionally rich blood supply is fundamental for the optimal functioning of islet endocrine cells. This represents a challenge for transplantation techniques to be successful.

### 1.3. Pancreatic Islets Isolation

**Figure 2:**
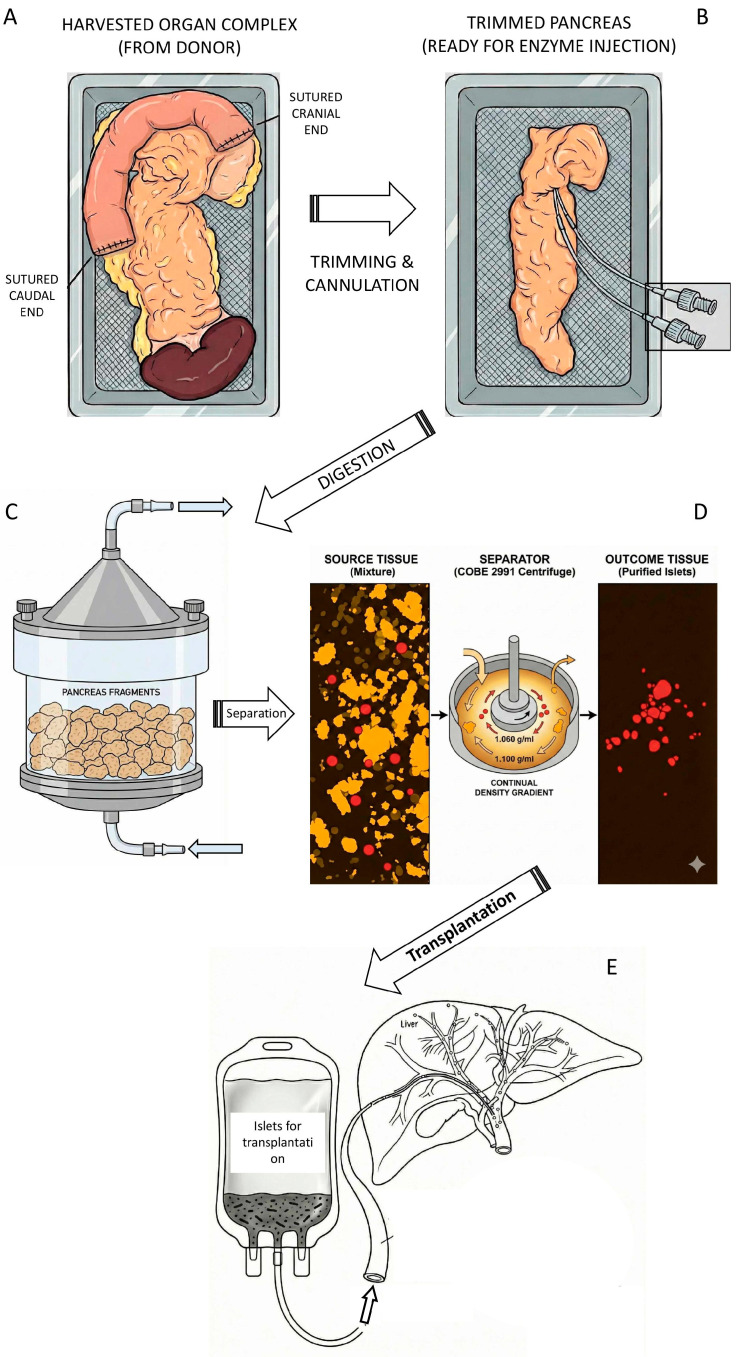
**Schematic illustration of islet isolation process** (this picture was created with the Gemini 3 Pro AI according to prompts of the corresponding author). (**A**) The pancreas was harvested from a cadaveric donor along with a segment of the duodenum and the spleen. To reduce the risk of contamination transfer from intestinal content, the duodenum was flushed with betadine solution and sutured at both ends. (**B**) The trimmed pancreas was cut in the neck segment and two catheters were inserted allowing instillation of enzymatic solution (mostly collagenase + neutral protease). (**C**) The distended pancreas was cut into small pieces and transferred to Ricordi chamber, a part of the digestive circuit. Under the cup of chamber there is a mesh with the holes 500 µm allowing circulation and collection of tissue pieces not larger than this size. (**D**) Collected tissue is spun in the density gradient (often Ficoll^®^) and islets are separated from exocrine tissue. The total tissue volume of 60 mL is typically reduced to 1–2 mL of purified islets. (**E**) Separated islets could be directly transplanted. The graft transferred to infusion bag is ready for transplantation. The catheter can be inserted in interventional radiology department through the 9th intercostal space to the portal vein.

Transplantation of isolated pancreatic islets represents the transfer process of healthy insulin- and glucagon-producing cells to diabetic patients. Islets intended for allogeneic transplantation are isolated from pancreases of non-diabetic cadaveric donors. As part of a multi-organ harvest, the pancreas is excised with duodenum and spleen together using the same technique as for organ transplantation. The duodenum is sutured on the sides, and the pancreas is perfused with a cold preservative solution. It is then transported in sterile conditions within chilled ice, see Figure 2A. Once it is accepted to the clinical isolation laboratory, the surrounding tissue is trimmed, and a catheter is inserted into the main pancreatic duct, see Figure 2B. A solution of collagenase and neutral protease enzymes is injected through the catheter. The pancreas is cut into several slices and placed into a “Ricordi chamber” to be gradually digested into small tissue pieces, see Figure 2C, releasing the islets from the exocrine tissue. The collected tissue is spun in a density gradient in order to separate the islets from the acinar tissue.

Autologous islets are isolated from non-diabetic patients who suffer from painful forms of chronic pancreatitis or other severe non-malignant disorders of the exocrine (acinar) tissue. They are typically isolated using a similar technique, but the main duct is often damaged, and the enzyme solution must be injected directly into the tissue. The need for density gradient centrifugation depends on total volume of collected tissue.

Islets are disconnected from the natural vascular network and a significant portion is lost during isolation [18]. According to the literature, multiple sites for islet transplantation have been tested: the subcapsular space of the kidney is advantageous due to its proximity to arterial blood supply but insufficient in terms of total volume for the graft insertion; the subcutaneous tissue offers minimal invasiveness but insufficient blood supply; the testicle offers immune protection but is not available in half of recipients; the peritoneal cavity offers virtually unlimited volume but insufficient blood supply without special preparation. Other sites such as anterior eye chamber or brain ventricles are totally experimental and not suitable for clinical use. The liver provides historically large experience, low invasiveness of the procedure, physiological drainage of insulin, and together with high concentrations of immunosuppressive drugs relatively nice probability of the graft immune tolerance. Currently, the portal vein system of the recipient’s liver provides the best outcomes. Therefore, it is the most commonly used transplantation site, and our review focuses on this graft location [19,20,21].

### 1.4. Pancreatic Islet Transplantation—Technical Aspects of Islet Insertion to Portal Vein

Transplantation of **allogeneic islets** into the liver is performed in principle by three approaches:

1. When transplanted with the kidney, the surgeon dissects out the peripheral branch of the portal vein in the mesentery and inserts a catheter under visual control, which minimizes the risk of bleeding and provides complete certainty of catheter placement.
2. In the case of islet transplantation alone, radiological catheterization is used. The catheter is inserted transcutaneously and transhepatically through the 9th-10th intercostal space, under ultrasound guidance through the peripheral branch of the portal vein into the main trunk, see Figure 2D [22]. Once the catheter is in place (verification by X-ray portography), the portal vein pressure is measured as an initial control level. Then the islets are inserted into the blood and spontaneously flow to the peripheral branches of the portal vein, where they settle. The direct contact of the graft with recipient blood induces a non-specific reaction, which is discussed below. After transplantation, the portal vein blood pressure is checked in order to indicate eventually the possible micro-thrombosis. When the tissue volume is large enough to be inserted in two steps (split into two bags), the portal vein pressure is checked after each infusion.
3. The rarely used alternative is laparoscopic recanalization of the umbilical vein [23,24].

In the case of **auto-transplantation**, the so-called second look operation is traditionally used, in which after several hours the surgeon reopens the patient’s abdominal cavity after total pancreatectomy, checks for potential sources of bleeding, and introduces the catheter into the main trunk of the portal vein under direct visual control [25]. The islets settle in the periphery of the liver and engraft there.

### 1.5. Primary Therapeutic Goals of Islet Transplantation

The primary goal of islet allo-transplantation is the restoration of the patient’s ability to reduce insulin secretion when glucose declines, thereby significantly reducing the risk of hypoglycemic coma. Sometimes when the graft is large enough (even combination of two or three grafts), it could release the patient from the need of daily insulin injections [26]. In the case of auto-transplantation the primary goal is to prevent the creation of new patients with extremely difficult-to-control diabetes as a result of therapeutic intervention. This provides much better diabetes control and in the long term a reasonable probability of preventing the development of long-term complications. Moreover, in 1/3 of cases, patients treated with islet auto transplantation remain free of exogenous insulin need. This fact makes islet transplantation a transformative therapy that is closer to a causal treatment than insulin injections are.

## 2. Liver as a Preferred Site for Islet Transplantation

### Anatomical and Physiological Features of Liver Location for the Islet Graft

Following insertion into the portal vein, pancreatic islets migrate downstream to smaller branches, where they become mechanically entrapped; 40% of small islets (up to 100 µm) can reach the sinusoids themselves (6–10 µm) [27]. Dual blood perfusion of the liver (arteries + portal vein) can prevent complete ischemia downstream of islet grafts. The high nutrient concentrations in portal blood impose a significant metabolic burden that can potentially damage the islets [28].

The initial cells interacting with the islets are the endothelial cells lining the branches of the portal vein and sinusoids. **Portal Vein Endothelial Cells (PVECs)** line continuously the portal vein and its branches keeping very low grade of permeability (lacking fenestrae). With more cuboidal shape they attach the continuous basal membrane and participate significantly in regulation and distribution of blood flow towards the liver. PVECs represent approximately less than 1% of the total liver cellular population. **Liver sinusoidal endothelial cells (LSECs)** are functionally highly specialized with unique morphological characteristics, such as the absence of basement membrane and the presence of open fenestrae that form a sieve-like structure [29,30]. Sinusoidal cells represent approximately 15–20% of liver non-parenchymal cells, yet occupy less than 3% of the liver’s volume [31].

Under physiological conditions, while PVECs serve as a vascular barrier, LSECs tightly regulate hepatic blood flow and maintain quiescence in hepatic stellate and Kupffer cells. In response to injury, including inflammation, PVECs can become more permeable in response to inflammatory stimuli or injury. This can lead to leakage of plasma proteins and fluid into the surrounding tissue. The presence of adhesion molecules on the internal surface can increase the secretion of chemokines and cytokines, which can amplify the inflammation. Some studies suggest the PVECs can transform into mesenchymal phenotype under specific circumstances, which contributes to liver fibrosis development. LSECs modify their phenotype through a process called capillarization. Cells lose their fenestrae, develop a basement membrane, and transform into continuous endothelium secreting anti-inflammatory IL-10, which ameliorates tissue damage and autoimmunity [32], and stimulates angiogenesis by VEGF secretion [30]. Sinusoidal cell dysfunction can activate hepatic stellate cells, promoting extracellular matrix deposition and fibrogenesis. They likely contribute to maintenance of IBMIR.

**Hepatocytes** are the dominant parenchymal cells representing almost 80% of all liver cells [31]. They have a unique structure, a hexagonal shape with several apical (canalicular) and basolateral (sinusoidal) plasma membrane domains [33]. The key role of hepatocytes is an uptake of nutrients, growth factors and other trophic agents, production of blood plasma proteins, maintenance of physiological blood chemistry, synthesis and transport of bile, breakdown of toxins, and contribution to innate immunity. Hepatocytes are considered one of the three most important insulin sensitive tissues, alongside muscle and adipose tissues.

During liver injury, hepatocyte release several cytokines, IL-6, IL-32, IL-33, and MCP-1, as well as extracellular vesicles containing exosomes, micro vesicles, or apoptotic bodies, facilitating crosstalk among surrounding cells [34]. Organelle damage particularly to mitochondria, lysosomes, and endoplasmic reticulum also occurs, contributing to inflammation and progression of chronic liver diseases. Inflammatory mediators like IL-1β, INF-γ, and TNF-α stimulate hepatocytes to produce nitric oxide (NO) [35], which affects oxidative metabolism, expression of target genes, glucose-stimulated insulin secretion, further DNA damage, and induces endoplasmic reticulum stress. Hepatocytes demonstrate very high capacity for tissue regeneration.

**Cholangiocytes** are a heterogeneous and highly dynamic population of epithelial cells lining the biliary ducts, accounting for approximately 3–5% of all hepatic cells. These cells exhibit pronounced polarity, with distinct apical (luminal) and basolateral plasma membrane domains. Under physiological conditions, cholangiocytes are predominantly quiescent and play a vital role in the modification of bile composition and volume. Based on anatomical location and functional characteristics, cholangiocytes can be classified into two main subtypes: small, minimally specialized intrahepatic cholangiocytes, which demonstrate relative resistance to hepatic injury and large, well-differentiated extrahepatic cholangiocytes possessing cilia, which are highly specialized but exhibit increased susceptibility to injury with reduced capacity of regeneration [36]. The cholangiocytes are preferentially blood perfused from liver arteries.

In response to injury or stress, cholangiocytes secrete cytokines (IL-1, IL-6, IL-8, and IFN-γ), nitric oxide, and growth factors which act in autocrine and paracrine manner and mediate cellular responses such as proliferation, apoptosis, senescence, angiogenesis, and fibrosis. Repair and remodeling of the biliary tree is supported by resident and recruited mesenchymal and endothelial cells. During acute injury, large cholangiocytes proliferate and maintain normal biliary homeostasis while during chronic injury, both types of cholangiocytes proliferate and induce reparative process.

**Kupffer cells (KCs)** are liver resident macrophages which are attached to the endothelial cells from the lumen side [37]; however, part of the cell body extends into the perisinusoidal Disse space where it interacts closely with stellate cells and hepatocytes [38]. They constitute 15% of all liver cells and 80–90% of all liver macrophages. Due to localization, Kupffer cells exhibit high phagocytic activity; they are the first macrophages exposed to various insults entering the liver via portal circulation. As a respond to transplantation injury, Kupffer cells are activated via complement pathway due to liver ischemia [39] and more specifically by the anaphylatoxins C3a and C5a, created during IBMIR [40,41]. When activated, KCs contribute to local inflammation and cellular destruction in two ways, through phagocytosis or by secreting inflammatory cytokines such as TNF-α, IL-1β, IFN-γ, nitrogen species [42], and coagulation factors, as well as tissue factor [43] and reactive oxygen species.

**Hepatic stellate cells (HSCs)**, previously known as Ito cells, lipocytes, or pericytes-like cells are liver-specific mesenchymal cells that play an important role in liver physiology and fibrogenesis. They are located in the space of Disse [37], represent 5–8% of liver cells, and physiologically they are quiescent, storing vitamin A in lipid droplets [37] and producing extracellular matrix (ECM) proteins. They contribute to immune tolerance, mediate intercellular communication, and trigger the synthesis of the plasminogen activation system components.

In pathological conditions following liver injury, hepatic stellate cells receive signals such as pro-inflammatory cytokines IL-1, IL-6, and TNF from the immune environment. Simultaneously, they can stimulate Kupffer cells, contributing to the inflammatory response. Additionally, HSCs exhibit potent immunosuppressive activity by promoting myeloid-derived suppressor cells [44] and inhibiting T-lymphocytes [45,46], thereby modulating the immune response during liver injury. Activated stellate cells lose lipid droplets with vitamin A and start to produce excessive ECM proteins associated with fibrosis and the loss of physiological architecture and function of the liver. A temporary scar at the site of injury is created to protect the liver from further damage, but if the scar becomes permanent this local liver micro-fibrosis may contribute to impaired islet graft function and survival [47].

**Liver dendritic cells (DCs)**, previously known as Pit cells, are professional antigen presenting cells capable of inducing proinflammatory immune responses as well as immune tolerance [48,49]. DCs are localized within the portal area attached to the endothelial cells and can be in contact with Kupffer cells [37]. Several subsets of DCs expressing different cell surface markers and cytokines with different antigen-presenting capability were identified in the liver. Plasmacytoid DCs (pDCs) are potent producers of IFNs. Myeloid DCs (classical cDC2s) are most abundant in the liver; they exhibit tolerogenic capacity and are the most potent producers of proinflammatory cytokines IL-6 and TNF-α. While lymphoid DCs (classical cDC1s) produce high levels of IL-12 [50].

After liver injury, DCs induce hepatic stellate cells, NK cells, and T cells to mediate inflammation. DCs also act fibrolytic and are involved in the regression of liver fibrosis. Interestingly, DCs are immature and tolerogenic in the steady state; in chronic hepatic injury they support inflammatory state and damage, and while in acute liver injury, they act as protective cells preventing structural damage. Mature DCs are detrimental for islet graft survival because of inducing an inflammatory cascade while immature ones are tolerogenic and are essential for islet allograft survival [51].

The main advantage of islet insertion into liver is the natural drainage of insulin into the hepatic parenchyma, which provides a “first pass” effect and reduces the burden of high insulin concentrations on the systemic circulation. Partial oxygen delivery via venous blood may also positively influence the graft. In the short-term, the oxygen supply is better than with some alternative techniques that simply implant islets in other sites (omentum, under skin, kidney capsule, etc.) but in the long-term perspective it is inferior to natural perfusion in pancreas.

Vascular density of transplanted pancreatic islets is comparable to that of the surrounding liver tissue [52]. However, several studies have reported that the vascular density of transplanted islets is lower compared to endogenous islets within the native pancreas [7,10,53,54,55,56]. Consequently, the blood perfusion of the grafts is less than 20% of the blood flow that perfuses native pancreatic islets, and it does not increase over time, up to 9 months post-transplantation [10,53,57,58].

Although single islet transplantation improves blood glucose level control, it does not always provide full normalization of glucose metabolism, which consequently keeps ß-cells maximally stimulated for insulin secretion. In combination with reduced vascular density, the significantly slower dilution and washout of insulin can be expected, leading to prolonged high local insulin concentrations, which may persist permanently and possibly enhance the insulin stimulatory effects on liver cells.

## 3. The Effects of Islet Transplantation on Liver

Considering the transplantation technique and the potential incompatibility between the islet graft and the hepatic environment, it is essential to evaluate the possible adverse effects across the early and delayed post-transplant periods. Many papers have been published summarizing a global outcome of transplantation, the effect of transplantation procedure itself on acute islet engraftment, and the long-term islet survival and function in an unnatural environment of portal vein. According to our best knowledge the paper reviewing the impact of islet transplantation on liver tissue has not yet been published. Therefore, this paper was prepared in order to summarize the early and delayed effects of pancreatic islets on surrounding liver tissue complimentary to other reviews, which were focused on islet grafts [59,60].

### 3.1. The Early Interaction of Liver with Islet Grafts

**Figure 3:**
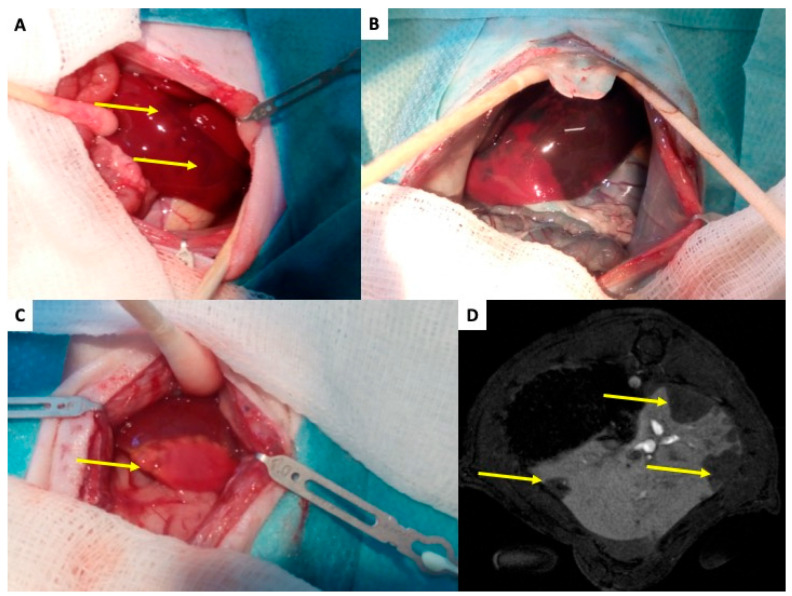
**Rat liver after transplantation of pancreatic islets—initial changes.** (**A**) The liver of islet recipient two hours after transplantation of 1000 islets into the portal vein. Yellow arrows indicate dark regions on liver surface, which represent tissues with impaired perfusion due to micro-thrombosis caused by islet graft and IBMIR. (**B**) The enhanced difference between tissue with normal (dark blue) and impaired (pink) blood perfusion. The Patent Blau^®^ (intravital staining marker) injected to the portal vein cannot enter tissue „behind islets” in de-arterialized liver model. (**C**) The liver with ischemic necrosis developed in two days after islet transplantation (white tissue in contrast to normal red-brown liver tissue)—indicated by yellow arrow. (**D**) MRI transversal slice covering the liver 2 h after transplantation of 1000 pancreatic islets. The arrow indicates regions without contrast agent, i.e., with impaired blood perfusion.

Following implantation into the portal vein, pancreatic islets migrate downstream to smaller branches, where they become mechanically entrapped. Larger islets (100–300 µm) are lodged at the level of the smaller portal veins (40–100 µm), and 40% of small islets (up to 100 µm) can reach the sinusoids themselves (6–10 μm) [27]. This obstruction results in a slow down or cessation of blood flow, microembolization, ischemia of the affected liver segment, and local activation of the immune response. The extent of tissue damage corresponds to the caliber of the occluded vein. Due to dual perfusion of the liver (artery and vein), complete ischemia does not occur mechanically; however, loss of portal vein perfusion distal to the graft causes significant hypoxia, which manifests as discoloration of the liver surface within minutes Figure 3A [59]. Intravital imaging with Patent Blau^®^ injected directly into the portal vein (two hours after islet transplantation) clearly showed areas of liver tissue devoid of coloration, indicating loss of portal perfusion. These areas later become stained blue due to arterial blood flow Figure 3B. By 48 h, ischemic necrosis developed in the tissue Figure 3C [61,62], which healed completely within a month [61,62]. In the rat de-arterialized liver model, contrast-enhanced magnetic resonance imaging (DCE-MRI) successfully visualized non-perfused tissue as early as 2 h after islet transplantation Figure 3D [61]. These morphological findings are accompanied with temporary increase in portal vein pressure [63,64] and liver functional tests [63,65]. Portal vein pressure is typically normalized in several hours and liver functional tests up to one week.

**Figure 4:**
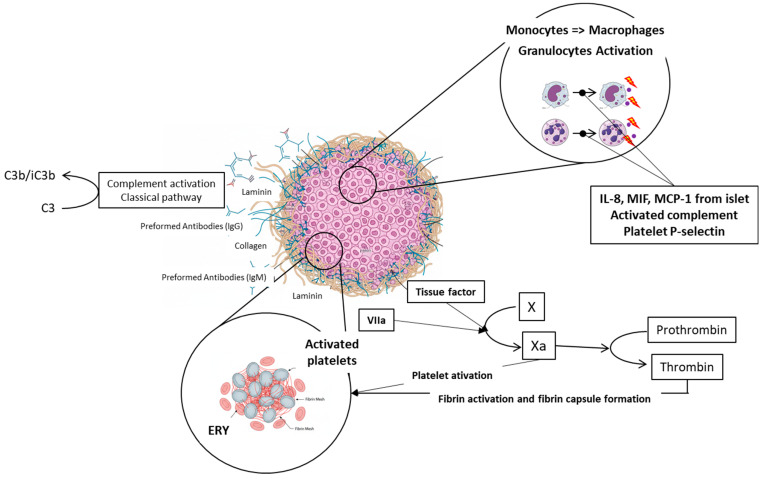
**Schematic description of IBMIR.** Natural immunoglobulins IgG and IgM bind to the collagen or laminin molecules on the islet grafts surface. This interaction triggers activation of complement and accumulation of C3b/iC3b fragments on the surface of islet cells, which stimulates inflammation. Parallelly, both coagulation pathways are activated; the extrinsic pathway is stimulated by tissue factor (TF) molecules on the surface of islet cells, and the intrinsic pathway is triggered by negative charge of islet surface. Individual parts of the picture were created with the help of AI Gemini Pro.

**Figure 5:**
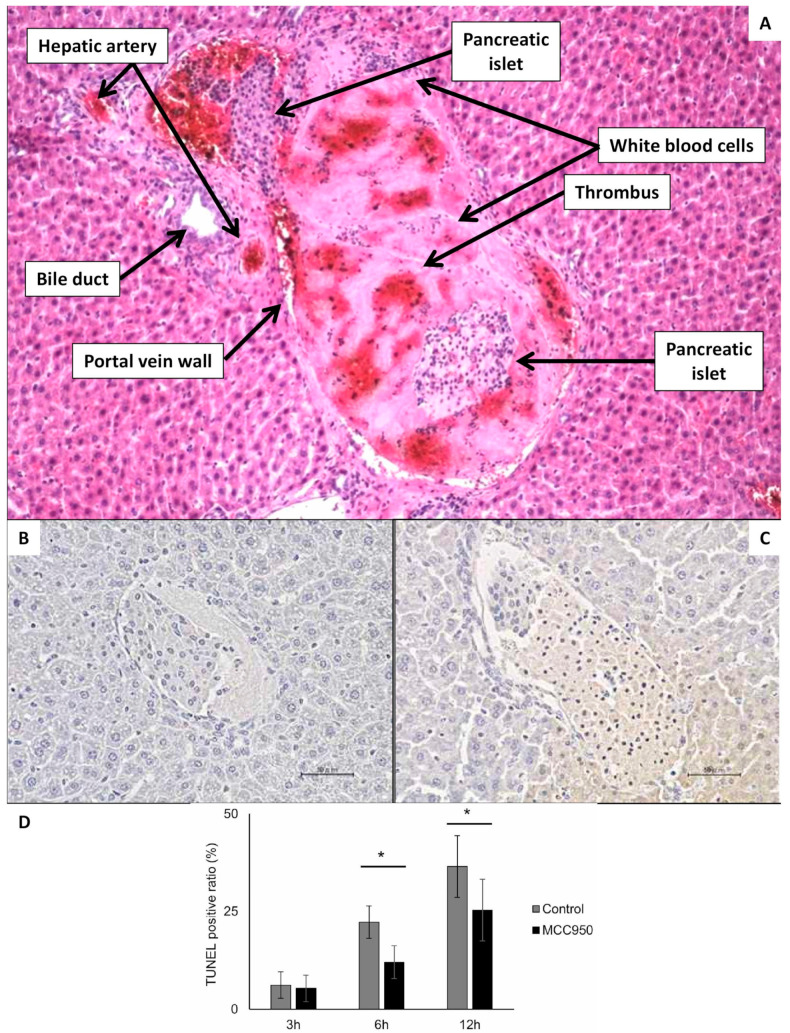
Microscopic visualization of islet interaction with portal vein blood and liver tissue several hours after transplantation. (**A**) Hematoxillin/Eosine staining of the liver 2 h after transplantation of pancreatic islets, which are entrapped within a thrombus fulfilling all the portal vein lumen. On the periphery of thrombus are visible white blood cells. The magnification ratio 100×. (**B**) Syngeneic islets 3 h after transplantation into portal vein of diabetic mouse. The section was stained using TUNEL. There are not any TUNEL-positive cells. (**C**) Syngeneic islets 6 h after transplantation into portal vein of diabetic mouse. The section was stained using TUNEL. There are frequent TUNEL-positive cells. (**D**) The chart indicating the ratio of TUNEL-positive cells in the three time points after transplantation of syngeneic islets with and without the therapeutic intervention with MCC950 substance (not significant for this review paper: the main purpose of using this image is to show that acute tissue hypoxia after transplantation causes significant apoptosis). Picture (**A**) is from the archive of authors (IKEM), and pictures (**B**–**D**) are reproduced from Figure 3 of Matsuoka paper [77]. * *p* < 0.05. (Scale bar 50 µm).

In addition to mechanical obstruction transplanted islets trigger several acute physiological responses in the portal vein and surrounding liver tissue including the endothelial injury, activation of the clotting cascade, platelet aggregation, the complement system activation, and immune cells recruitment. These responses are collectively known as the Instant Blood Mediated Inflammatory Reaction (IBMIR, see Figure 4). Once the islets are infused into the portal vein blood, the natural immunoglobulins (IgG and IgM) bind mainly to the collagen or laminin molecules on the surface of the islets. These molecules are not normally in direct contact with the blood [66]. This interaction subsequently activates the complement cascade, resulting in the accumulation of C3b/iC3b fragments on the surface of islet cells. This accumulation serves as a stimulus for inflammation. Parallelly, both coagulation pathways are activated; the extrinsic pathway is stimulated by tissue factor (TF) molecules on the surface of islet cells [67], and the intrinsic pathway is triggered by negative charge of islet surface in general [68]. TF’s interaction with factor VIIa activates factor X, converting prothrombin to thrombin, which directly activates platelets (increase in the GPIIb-IIa and a2b1 integrins’ affinity), leading to fibrin capsule formation around islets [40,67]. Cytokines released from islets (IL-8, MIF, MCP-1, etc.) activated complement and P-selectin in activated platelets recruits neutrophils and monocytes, resulting in islet infiltration and severe damage [69,70], see Figure 5. Up to 50–60% of transplanted islets can be destroyed. The sinusoidal endothelium, necrotic exocrine tissue contaminating the islet graft [71], and endotoxin contaminant of the islet graft originating from collagenase digestion [72] also contribute to stimulation of Kupffer cells.

While the portal vein micro-thrombosis and mild hypertension followed by engraftment into the vessel wall dominates in humans and large animals [52,73], more significant tissue ischemia, necrosis, and full endothelial disruption are dominant in rodents, likely due to different islet/portal vein diameter ratio [52]. Therefore, in the human liver, the majority of transplanted islets are found within the portal vein lumen or incorporated in the portal vein walls [74,75]. In small animals’ liver, transplanted islets are primarily found embedded within the liver parenchyma [76]. The isolation process injures islets as well as reperfusion reaction, but the transplantation into portal vein triggers a reaction of liver tissue as well [59].

If summarized, the insertion of islet grafts into the portal vein and their embolization in the corresponding caliber of smaller branches leads to mechanical obstruction of blood flow. Direct contact with blood causes activation of clotting cascade and non-specific inflammatory mechanisms, which damage both the graft itself and the surrounding liver tissue. Repair processes follow, leading to complete healing of the liver tissue and loss of approximately 50–60% of the graft cells. Depending on the extent and severity of damage to the vascular wall and associated liver tissue, the graft is finally located either in the vascular wall with reendothelialization, in the case of minor damage, or in the liver parenchyma with subsequent repair of vascular structures, in the case of severe damage and local complete destruction of the vascular wall. Severe portal thrombosis is a very rare complication, while micro-thromboses accompanied by a temporary increase in portal pressure and elevated liver function tests are common.

### 3.2. The Delayed Interaction of Liver with Islet Grafts

Insulin plays dual roles as a metabolic regulator and a growth factor. Once the islet engraftment is complete, the immediate and long-term effects of high insulin levels on surrounding liver tissue are especially relevant. In the pancreas, the insulin concentration inside the islet blood is approximately 1000 times higher than in the systemic circulation. This difference can affect the morphology of the surrounding pancreatic acinar tissue. Cells adjacent to the islets exhibit multiple nuclei and increased number of zymogenic granules. [15,17,78]. Similar morphological changes have been reported in the liver, where a band of steatotic hepatocytes surrounds the insulinoma metastases, which was confirmed by the magnetic resonance imaging, CT, ultrasound, and also histologically [79]. The same kind of changes have been described in diabetic patients suffering from chronic kidney failure, who were treated by peritoneal dialysis (solution containing simultaneously both glucose and insulin). Magnetic resonance imaging has shown the hypodense sub-capsular streak, characterized histologically as the liver steatosis [79].

In case of islet transplantation, four months after the first pancreatic islet transplantation to his patient, Eckhard et al. identified small hypodense hepatic lesions by ultrasound [80], which were subsequently confirmed by MRI and histological examination as fatty degeneration. These benign morphological changes were attributed to local hyperinsulinemia in the vicinity of transplanted islets. Similar periportal steatosis was later reported by Markman and Toso [73,81]. Importantly, these alterations were observed predominantly in recipients with partial graft function who remained slightly hyperglycemic after transplantation [73,80]. In these patients, sustained hyperglycemia maintained maximal β-cell stimulation, leading to chronic local hyperinsulinemia. By contrast, recipients with full graft function and normoglycemia showed no hepatic alterations during four years of follow-up [73,74]. Collectively, these findings indicate that persistent hyperglycemia and associated hyperinsulinemia are key drivers of post-transplant liver steatosis and glycogenosis.

Comparable liver changes were documented experimentally in diabetic rat models, where steatosis and glycogenosis were confined to the hepatic lobes receiving intraportal islet transplants [82,83,84,85]. Histological analyses revealed hypertrophic hepatocytes adjacent to transplanted islets, characterized by abundant glycogen and lipid accumulation, supporting the hypothesis that local hyperinsulinemia mediates these alterations [84]. In nonhuman primates, hepatocellular glycogenosis was also observed in the vicinity of intrahepatic islets. Notably, only the primate monitored for seven months exhibited morphological changes consistent with glycogen storage [86].

In order to clarify the molecular background, mechanistic studies have highlighted alterations in the insulin-like growth factor (IGF) pathway within glycogen-storing hepatic foci [83]. Specifically, IGF1 and IGFBP4 were upregulated, while IGFBP1 was downregulated. Findings from primary hepatocyte cultures corroborated these results, demonstrating that insulin strongly suppresses IGFBP1 expression while upregulating IGF1 and IGFBP4 [87,88]. These changes in IGF–IGFBP gene expression provide strong evidence of local hyperinsulinemia-induced modulation of hepatocyte signaling after transplantation [89]. As has been already mentioned, insulin/IGFs activate the external α units of the tyrosine kinase of the appropriate receptors (IRs and IGF1R). This initial binding causes a conformational change, activating the kinase domain of the intracellular β subunits. Which leads to tyrosine phosphorylation of insulin receptor substrates (IRSs). In the liver, phosphorylation of IRS2 is dominant over phosphorylation of other IRSs [90,91,92,93,94]. Phosphorylation of IRSs leads to activation of two major post-receptor signaling pathways.

**The first one** (Akt/mTOR pathway-mainly metabolic) triggers via phosphatidylinositol 3-kinase (PI3K). PI3K induces activation of the Akt-kinase. Akt-kinase subsequently activates a variety of downstream signaling kinases, including the mammalian target of rapamycin (mTOR). Through these signaling kinases, Akt stimulates lipogenesis, regulates glycogen synthesis, inhibits gluconeogenesis, and regulates cell growth. Activated mTOR molecule stimulates protein synthesis and inhibits proteolysis and apoptosis [90,91,92,93,94].

**In the second** cascade (ERK pathway, mainly mitogenic) phosphotylated IRS proteins bind Grb2/son of sevenless (SOS) complex thereby activating Ras/Raf/MEK/mitogen activated kinase (MAPK) complex. The last member of this branch, the ERK, is moved to the cell nucleus, where it stimulates the cell proliferation and differentiation [90,91,92,93,94]. Additional insights were provided by Evert et al. [85], who reported enhanced insulin signaling following transplantation, manifested by elevated insulin receptor (IR) expression and overexpression of multiple Ras–Raf–MAPK–ERK pathway proteins, including IRS-1, Raf-1, and Mek-1. Given the low IGF1R expression in these lesions, signaling was most likely mediated through IR rather than IGF1R [82].

Taken together, clinical and experimental evidence indicates that hepatic steatosis and glycogenosis after intraportal islet transplantation are a consequence of increased local insulin action. This effect arises from the combined influence of persistent hyperinsulinemia, mild hyperglycemia, and immunosuppression-induced alterations in the portal microenvironment [80,81].

The cells with lipid and glycogen deposits are called clear cell foci [95,96,97,98], which can later transform to cystic cholangiomas, hepatocellular adenomas, and carcinomas in several animal models [99,100,101,102,103]. The specific contribution of insulin release or toxic effect of diabetes inducer is still not fully clear. Observations implicate altered insulin/IGF signaling—particularly increased IGF2 bioavailability, elevated IGF1 receptor expression, and activation of the AKT/mTOR pathway—in lesion development and growth [83,85,96,98,104,105]. Although the administration of streptozotocin (STZ), a commonly used diabetogenic agent that destroys ß-cells, has been proposed as a significant cofactor, experimental designs using autoimmune diabetic models suggest that local hyperinsulinemia adjacent to islet grafts plays a primary role [83,85,96,98,104,106]. Further studies are required to define mechanisms and clinical relevance.

In the late 1970s, several studies reported the occurrence of cystic liver lesions following intraportal islet transplantation in rats made diabetic by a single streptozotocin (STZ) administration or pancreatectomy [99,100,101,102,107]. These cystic lesions in the liver were characterized by a lining of cuboidal epithelium typical for biliary tree. A permanent but not entirely widespread debate continues regarding the etiology of these liver lesions. One group of researchers hypothesized that local hyperinsulinemia associated with the transplanted islets might evoke the formation of liver cysts [100,107]. Conversely, another group postulated that the induction of diabetes via STZ might play a crucial role in the development of these liver cysts [99,101,102].

**Figure 6:**
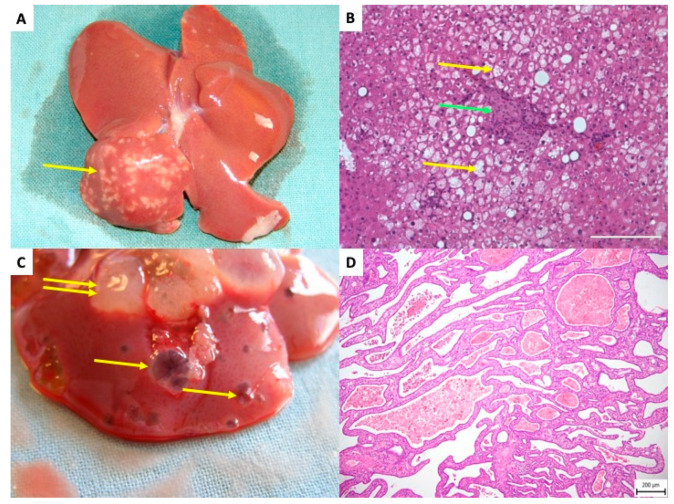
**Rat liver after transplantation of pancreatic islets–delayed side effects.** (**A**) The white dots in right liver lobes are related to a focal steatotic lesions spatially connected to islet grafts—10 months after transplantation. Yellow arrow indicates the focal steatotic lesion. (**B**) Detail microscopic examination of the same lesion as in (**A**). The yellow arrow indicates so-called foam cells (hepatocytes full of lipid particles). Green arrow indicates islet graft. (**C**) Macroscopic view of cystic liver lesions surrounding transplanted islets. Single yellow arrows indicate simple cysts; double arrows indicate complex polycystic lesions. (**D**) Microscopic picture, Hematoxillin Eosine. Polycystic lesion showing septa with cuboid cholangiocyte epithelial cells outlining the lesions.

Kříž et al. observed the onset of cystic changes in the liver as early as six months post-transplantation of pancreatic islets in insulin-sensitive Brown Norway (BN) rat strains [105]. These cystic alterations presented either as simple cysts or as cystic adenomas lined by a single layer of flat or low cuboidal epithelium. The authors proposed that STZ might trigger subsequent cholangiocellular lesions Figure 6.

Evert et al. wanted to investigate the impact of islet transplantation on adjacent bile duct epithelia in diabetic rats and to assess the influence of STZ. Their study utilized two distinct inbred rat strains: Lewis rats, in which diabetes was induced by STZ, and autoimmune diabetic Biobreeding (BB) rats, which served as a model for autoimmune type 1 diabetes mellitus and naturally developed high blood glucose levels without STZ induction. The follow-up duration spanned from 6 to 24 months. Cystic lesions developed to varying extents in all diabetic animals receiving low-number intrahepatic islet transplants, including both autoimmune BB rats and STZ-induced diabetic Lewis rats. Of particular note, BB rats with autoimmune diabetes exhibited a significantly higher incidence of tumor-like cystic lesions compared to control groups. Nonetheless, liver lesions were more prevalent in recipients with STZ-induced diabetic Lewis rats than in autoimmune BB rats. In this model STZ contributes by an initial injury on the formation of cholangiocellular cysts to an effect that can be amplified by insulin. It could be speculated that STZ as an alkylating agent can damage DNA or some nuclear protecting mechanisms, which allows full “expression” of insulin overstimulation in liver cells. However, the impact of post-transplantation hyperinsulinemia appears to hold greater significance than the modest influence of STZ. Immunohistochemical analyses corroborate that local hyperinsulinemia promotes cholangiocellular proliferation, leading to benign cystic cholangioma development. This is marked by a pronounced overexpression of intracellular proteins involved in the Ras–Raf–MAPK pathway [98].

Numerous long-term rodent studies have demonstrated that pancreatic islet grafts are associated with the development of hepatocellular neoplasia [83,85,96,104]. In experiments conducted by Scharf, Dombrowski, and colleagues, PI were transplanted selectively into the right hepatic lobe, while the contralateral left lobe served as an intraindividual control. Hepatocellular adenomas (HCAs) and hepatocellular carcinomas (HCCs) were identified between 15 and 22 months post-transplantation, exclusively within the graft-bearing (right) lobes [83,96,104]. In one series, at least one HCA was observed in 86% of recipients, whereas HCC was documented in 19% of cases [104]. While small glycogen-storing foci were observed in some healthy animals after 24 months, neoplastic lesions such as HCAs or HCCs were not detected in these controls.

Histopathologically, HCAs extended beyond the original hepatic acini, exhibiting sharp boundaries and compressing the surrounding parenchyma [96]. HCCs were defined as tumors larger than 5 mm in diameter, characterized by trabeculae thicker than three cell layers in at least two distinct regions, along with a high mitotic index, vascular invasion, or metastatic spread [96].

The potential synergistic carcinogenic effects of STZ were controlled through appropriately designed experimental groups, excluding a confounding role for STZ in tumor development [104]. Several investigations have addressed alterations in the insulin/insulin-like growth factor (IGF) signaling pathway implicated in liver neoplasia [95,108,109,110]. Specifically, analyses by Scharf et al. and Dombrowski et al. examined various components of this pathway in the context of islet transplantation [83,96,104].

Immunohistochemical analyses revealed that hepatocellular carcinoma (HCC) tissues post-transplantation exhibited reduced IGF1 expression compared to adjacent non-tumorous liver tissue; however, some tumors demonstrated significant IGF2 overexpression [83]. Furthermore, decreased local production of insulin-like growth factor binding proteins (IGFBPs) following islet transplantation was observed, which may contribute to IGF2 overexpression [83,84]. These alterations in IGFBP levels, by modulating IGF2 bioavailability, appear central to the development and progression of HCC.

During hepatocarcinogenesis, an increase in insulin-like growth factor 1 receptor (IGF1R) expression has been documented as lesions progress from preneoplastic foci to overt HCC [83,89]. Preneoplastic hepatic foci typically display minimal IGF1R expression, with insulin interacting with the insulin receptor (IR), whereas in established HCC, elevated IGF1R expression facilitates the interaction with IGF2, promoting tumor growth.

These findings align with broader research on HCC, which demonstrates that IGFBP expression is markedly downregulated in human HCC tissues [108,109] and that IGF1R is widely expressed across various human malignancies [108]. Cell proliferation in both HCAs and HCCs, as measured by BrdU labeling, was significantly enhanced compared to non-neoplastic parenchyma [83,96,104]. Two primary insulin signaling pathways implicated in hepatocarcinogenesis are the Ras-ERK pathway and the Akt/mTOR pathway [110,111]. Following intrahepatic islet transplantation, the Ras-ERK pathway was downregulated, evidenced by decreased expression of IRS-1, Raf-1, and MEK-1 [83]. Consequently, the Akt/mTOR pathway is posited as the principal driver of cellular proliferation observed in this context. Evert et al. further confirmed the critical role of the Akt/mTOR signaling cascade in HCC development post-transplantation [112]. In summary, the carcinogenic process was confined to the hepatic lobe containing the islet grafts. Downregulation of IGFBPs led to IGF2 overexpression, which subsequently interacted with upregulated IGF1R, activating the Akt/mTOR pathway and promoting tumorigenesis.

The clinical significance of this phenomenon is highlighted by the higher incidence of cancer in patients with type 2 diabetes mellitus, which is characterized by tissue insulin resistance and the resulting compensatory hyperinsulinemia [113,114]. Permanent hyperinsulinemia leads to the increased activity of synthetic and mitogenic arms of insulin signaling cascade, transmitting the growth factor effect of insulin. Overstimulated mitogenic arm of insulin signaling cascade is considered to be an effective enhancing mechanism in pathogenesis of hepatocellular carcinoma in type 2 diabetic patients [113,114]. The incidence of hepatocellular carcinoma is approximately twice as high in these patients compared to the healthy population [115,116]. Intrahepatically transplanted pancreatic islets cause local hyperinsulinemia, whose stimulatory effect could be even more pronounced. But the reported cholagiogenic cystic lesions or malignant tumors related to islet grafts were never reported in human patients.

## 4. Conclusions

Transplantation of isolated pancreatic islets into the portal vein causes tissue damage during both the acute and long-term periods. Initially, tens of minutes after transplantation, mechanical obstruction of small branches of the portal vein occurs, which is further exacerbated by local micro-thrombosis. This leads to focal hypoxia, necrosis, inflammation, damage to the endothelium, and damage to the integrity of the entire vascular wall. Subsequently, healing processes are activated, resulting in the attachment of approximately half of the administered islet graft to the wall of the portal vein branches in large animals and humans. In contrast, in small animals, the integrity of the vascular wall is completely disrupted and most of the islets engraft directly to the liver parenchyma outside the portal vein branches. In several weeks or months after transplantation focal steatosis and glycogenosis (clear cell foci) develop in hepatocytes surrounding islet grafts. Later on, some clear cell foci can develop in cystic cholangiogenic complexes and rarely to hepatocellular carcinoma. However, this phenomenon was observed only in experimental animal models and likely arises from combined assumptions, the initial injury of liver cells by streptozotocin, and permanent hyperinsulinemia surrounding islet grafts. Importantly, cystic cholangiomas or hepatocellular carcinomas related to transplanted islets were never reported in human patients.

To summarize, the currently available data indicate that islet transplantation to liver of human diabetic patients despite being associated with temporary side effects, is generally considered safe in the long-term perspective.

## Figures and Tables

**Table 1:** Basic summary of large pancreatic islet composition.

Cell Type	Hormone	Percentage
α (alpha) cells	Glucagon, GLP-1	15–20%
β (beta) cells	Insulin	65–80%
δ (delta) cells	Somatostatin	3–10%
γ (PP) cells	Pancreatic polypeptide	3–5%
ε (epsilon) cells	Ghrelin	1%
Other cell types:	Stromal cells, blood cells, immune cells, neurons, endothelium	

## Data Availability

No new data were created or analyzed in this study. Data sharing is not applicable to this article.

## Abbreviations

The following abbreviations are used in this manuscript:

IR	Insulin receptor
IGF1R	Insulin like growth factor receptor
PI	Pancreatic islet
Tx	Transplantation
IBMIR	Instant Blood Mediated Inflammatory Response
ECM	Extracellular matrix
PVECs	Portal vein endothelial cells
LSECs	Liver sinusoidal endothelial cells
KC	Kupffer cells
HSC	Hepatic stellate cells
DC	Dendritic cells
HCA	Hepatocellular adenoma
HCC	Hepatocellular carcinoma
